# Polysorbate 80 and histidine quantitative analysis by NMR in the presence of virus‐like particles

**DOI:** 10.1002/elps.202100189

**Published:** 2022-04-26

**Authors:** Richard R. Rustandi

**Affiliations:** ^1^ Analytical Research Development Merck & Co., Inc. Kenilworth New Jersey USA

**Keywords:** histidine, HPV, NMR, polysorbate‐80, stainless steel, vaccine

## Abstract

Polysorbate‐80 (PS80) and histidine are common excipients in vaccine and therapeutic protein formulation. A simple quantitative NMR method to measure both PS80 and histidine in human papillomavirus (HPV) virus‐like particle (VLP) vaccine for aqueous and alum‐containing samples is described. The new NMR method is compared to current colorimetric methods for PS80 and RP HPLC for histidine. The new NMR method is comparable to current assays with an advantage of a simpler sample treatment for PS80. The efficiency is also increased because one method can now provide two assay results instead of two separate methods. Furthermore, the NMR method can detect PS80 stability due to hydrolysis and oxidation when PS80 is stored in a stainless steel container by observing a change of its NMR line shape profile.

**Abbreviations**
HPV: human papilloma virus
PS80: polysorbate‐80
TP: therapeutic protein
UV: ultraviolet.

## 1 INTRODUCTION

Human papillomavirus (HPV) is a virus that its infection can cause several types of cancer such as cervical, anal, oropharyngeal, penile, vulvar, vaginal, as well as genital warts [1]. The current HPV vaccine, Gardasil^®^9, which protects against nine types of HPV, is a virus‐like particle (VLP) capsid formed by 72 pentameric capsomers of HPV structural protein, L1 (55 kDa) [2]. The vaccine final aqueous bulk formulation contains histidine buffer, polysorbate 80 (PS80), and sodium chloride, while the drug product contains additional alum adjuvant. During the vaccine development, an NMR spectroscopy method was developed to analyze both histidine and PS80 in the presence of HPV VLP.

PS80, or Tween 80, and histidine buffer are two of the most common excipients used in formulations for vaccine and therapeutic protein (TP) products. While the function of histidine is as a buffer for maintaining pH, PS80 has dual roles in preventing protein surface adsorption and induced aggregation due to process of agitation, stirring, shaking, freezing or thawing, as well as lyophilization [3]. PS80 is a nonionic surfactant and has attractive physical properties such as a low CMC, high hydrophilic‐lipophilic balance, and low toxicity for biological applications in vaccines or TPs [4].

**FIGURE 1:**
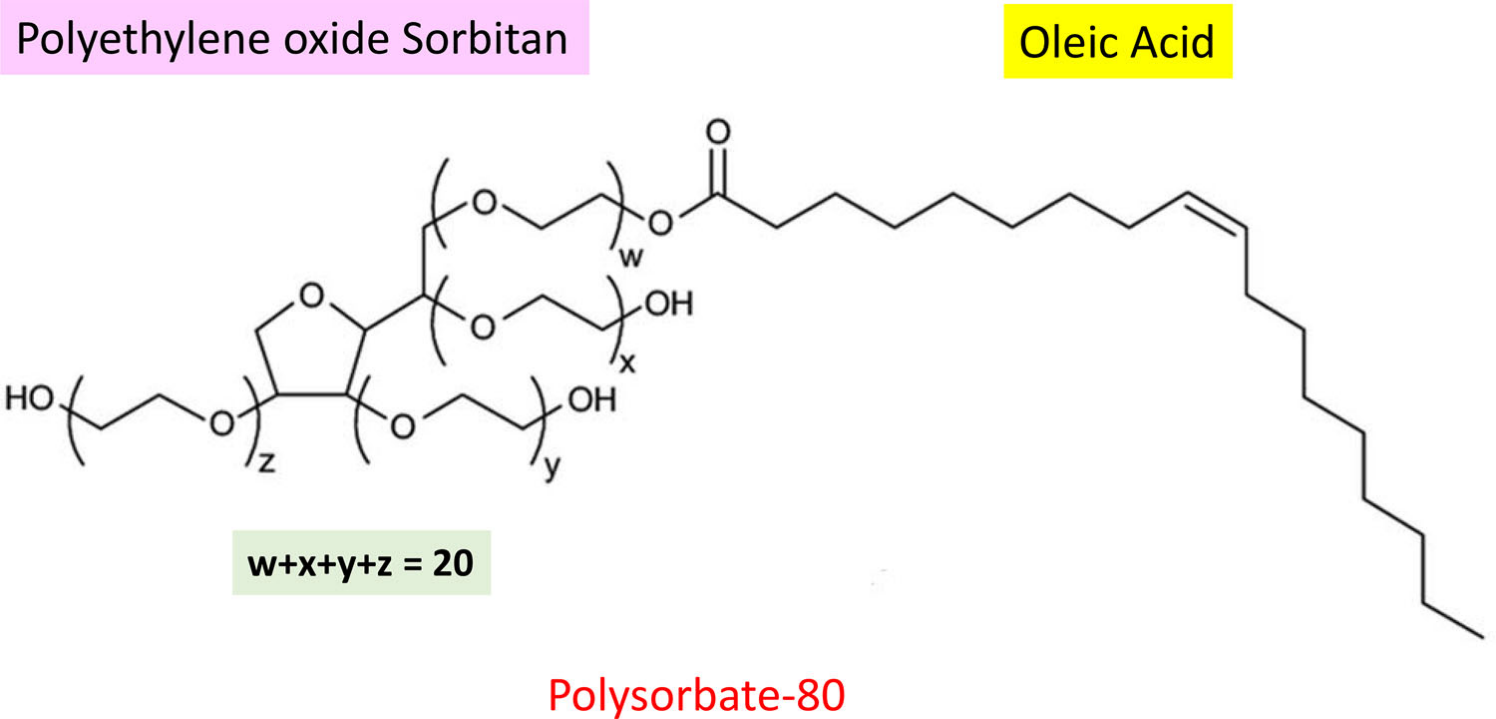
Polysorbate‐80 structure

PS80 is composed of fatty‐acid esters where various lengths of fatty‐acid hydrocarbons provide its hydrophobic feature, whereas its hydrophilic feature is due to the lengths of ethylene oxides attached to sorbitan (Figure 1). Oleic acid is the most abundant fatty acid found in PS80, followed by various other fatty acids such as linoleic acid, palmitic acid, palmitoleic acid, stearic acid, myristic acid, and linolenic acid [5]. Due to the structural and heterogeneity complexity of PS80 and its importance in vaccine and TP formulation, it requires many different analytical tools to quantitate, analyze, and measure stability. As a result, there are several PS80 analytical methods that have been reported in the literature. For example, simple colorimetric assays using ammonium cobalt thiocyanate that detects polyethylene oxide [6] and iodine‐starch detecting polyoxyethylene stearate [7]. There is also a plate‐based assay using fluorescent detection to detect the micelle form of PS80 [8]. Another indirect method is detecting the oleic fatty acid after acid hydrolysis using GC [9] or HPLC [10, 11]. In addition, many direct detection methods of PS80 using HPLC have been reported in the literature. They mostly employ evaporative light scattering detectors [12, 13], charged aerosol detectors [14, 15], as well as MS [16, 17] detectors to increase sensitivity and evaluate their degradation products.

Another direct method of PS80 quantitation is NMR spectroscopy. There are few reports in the literature describing the use of NMR for PS80 analysis [18, 19, 20]. Most reports characterize PS80 by NMR in the absence of protein or particles. Recently, Poppe et al. describe hydrodynamic profiling by NMR (HAP‐NMR) in the presence of high concentrations of mAbs, however, this method can only estimate the PS80 concentration [21]. Here, we report a much simpler ^1^H NMR method in the presence of HPV VLP using standard curves. Furthermore, the method is capable of detecting PS80 profile changes when it is stored in stainless steel containers. In addition, the current NMR method can also be used to analyze the histidine buffer concentration in HPV VLP samples in the same experiment.

## 2 MATERIALS AND METHODS

### 2.1 Chemicals and reagents

PS80 was purchased from Pierce (Rockford, IL, USA) or Croda Inc. (Plainsboro, NJ, USA). l‐histidine and NaCl were bought from Sigma (St. Louis, MO, USA). D_2_O (99.9%) and sodium 2,2‐dimethyl‐2‐silapentane‐5‐sulfonate‐*d*_6_ (DSS‐*d*_6_) (2%) were purchased from Cambridge Isotope (Andover, MA, USA). NaOD was purchased from Isotec/Sigma‐Aldrich (Miamisburg, OH, USA).

### 2.2 HPV VLP L1 preparations

The L1 protein for all HPV types were expressed in yeast (*Saccharomyces cerevisiae*) and their purification steps have been described previously in published procedures [22, 23]. All L1 proteins have purity of more than 95% based on the reducing SDS‐PAGE gel with Coomassie staining. The purified L1‐protein concentration was determined using a commercial bicinchoninic acid protein assay kit from Pierce.

### 2.3 NMR spectroscopy

Five standard samples were prepared to make a calibration curve, each standard containing both PS80 and histidine in 0.5 M NaCl. The calibration curves ranged from 50 to 800 µg/mL for PS80 and 2.5 to 40 mM for histidine (pH 6.2). HPV VLP sample (400 µL) was also prepared similarly. All standards and samples were lyophilized for 6–12 h in the speed vac (Savant SC110A). The dried standards and samples were resolubilized in 792 µL D_2_O and 8 µL DSS‐*d*_6_ as an internal chemical shift reference standard. They were spun down and transferred to a 5 mm NMR tube from Wilmad (Vineland, NJ, USA). The NMR experiment was done with Varian Inova 600 MHz equipped with Varian 5 mm 1H{13C/15N} PFG high‐field triple‐resonance room temperature probe using 1D proton 1‐pulse with two steady‐state scans, 3.0‐s acquisition time, 20‐s recycle delay, and 16 scans. The total experiment time for each sample was approximately 6 min. The sample temperature was maintained at 25°C during experiment. To eliminate a potentially small difference in buffer concentration for the reference standard and the sample during preparations, the magnetic field shimming was performed, line shapes were checked, and the proton 90° radio frequency pulse (9.25 µs) was calibrated for each reference standard and sample tested. The NMR spectrum was acquired with a 6999.7 Hz spectral width which covers from –1 to 10.6 ppm. The data were Fourier transformed to 42 K data points with 0.5 Hz line broadening. Chemical shifts were referenced to internal DDS‐*d_6_* as 0.00 ppm. The NMR data were processed using standard procedure with the peak intensities of 3.70 ppm for PS80 and 7.21 ppm for histidine integrated and used to plot the standard curve. The PS80 and histidine concentrations were calculated by comparing them with the standard curve. The standard curve was generated using linear regression and the slope and *y*‐intercept were obtained.

### 2.4 Colorimetric PS80 assay

The colorimetric PS80 method is described in the publication of Cucakovich [7]. The methodology is based on the formation of a complex between the PS80 polyoxyethylene stearate and the amylose fraction of potato starch. The free amylose combines quantitatively with iodine to form a blue amylose‐iodine complex with peak absorbance at 605 nm. Increasing PS80 will decrease starch‐iodine (blue color complex) interaction, thus, decreasing the absorbance at 605 nm. Plotting PS80 concentration with absorbance at 605 nm provides a linear relationship from 2.5 to 30 µg/mL PS80 range (0.00025 to 0.003%, w/v) of PS80. Briefly, the assay was done by adding 1 mL sample or PS80 standard and 1 mL acidified starch solution into a test tube and incubated 30 min at room temperature. Subsequently, 1 mL of I_2_ (0.005%)/KI (0.01%) solution was added into this mixture and incubated for 20 min at room temperature, and absorbance at 605 nm was recorded using Beckmann DU‐7400 ultraviolet (UV)/Vis spectrophotometer.

### 2.5 HPLC histidine assay

The quantification of histidine in HPV VLP samples used a RP‐HPLC employing a Phenomenex Luna 5 µm C18 RP 4.6 × 150 mm column. The isocratic mobile phase of 10 mM phosphate, 0.5 M NaCl buffer was used with a 0.5 mL/min flow rate, 50 µL injection, UV detection at 220 nm, and a 7‐min run time. In this procedure, a histidine standard curve (31.3–500 µM) is generated by regressing histidine peak areas against the known histidine concentrations. A histidine concentration value for each sample is calculated using the standard curve parameters.

## 3 RESULTS AND DISCUSSION

### 3.1 Polysorbate 80 and histidine in HPV VLP

**FIGURE 2:**
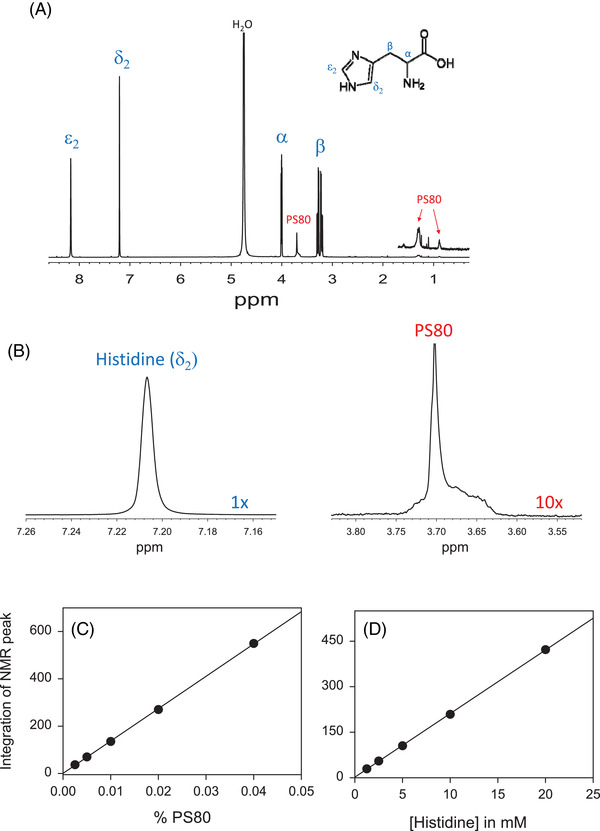
(A) NMR spectrum of PS80 and histidine in the presence of HPV VLP samples illustrating that no HPV VLP is observed; (B) the enlarged of PS80 and histidine peaks at 3.70 and 7.21 ppm, respectively used for quantitation. (C) Standard curve for histidine. (D) Standard curve for PS80

The NMR spectrum of HPV VLP sample in the final aqueous product containing PS80, histidine, NaCl, and HPV VLP at pH 6.2 was collected in D_2_O (Figure 2A). It shows the PS80 peak at 3.70 ppm that belongs to the polyoxyethylene (–O–CH_2_–CH_2_–O–) group, while peaks at 1.3 and 0.97 ppm are from CH_2_ and CH_3_, respectively, in fatty‐acid chain. Other PS80 protons, such as double bonds in oleic acid at 5.3 ppm, are very weakly observed due to low intensity at this concentration. All NMR histidine peaks of α‐proton (4.0 ppm), β‐proton (3.2–3.3 ppm), δ_2_‐proton (7.21 ppm), and ε_2_ proton (8.2 ppm), are observed. No protons from L1 protein in HPV VLP are detected as VLP is very large (MW ∼20 MDa), hence, it gives fast T2 relaxation. This similar principle could be applied to a smaller protein such as a mAb (∼150 kDa). However, the signal peaks from the mAb could be detected due to its high concentration (50–100 mg/mL) and slower T2 relaxation compared to VLP and could potentially overlap with the signal from formulation buffer matrix PS80 and histidine. Figure 2B shows the enlarged area of δ_2_‐proton (7.21 ppm) of histidine and polyoxyethylene protons (3.70 ppm) of PS80 which show a broad shoulder peak which is likely due to the micelle form of PS80. These two peaks are used for quantitation analysis of histidine and PS80, respectively.

To quantitate the amount of PS80 and histidine in the HPV final aqueous product, standard curves of PS80, ranging from 0.0025 to 0.04% (w/v), and histidine ranging from 1.25 to 20 mM, were prepared and their NMR spectra were collected in D_2_O. Peak area at δ = 3.70 ppm for PS80 (integration range from δ  = 3.78–3.58 ppm) and δ_2_ proton in histidine at δ = 7.21 ppm (integration range from δ  = 7.25–7.15 ppm) were used for peak area integration; the %RSD was calculated for five independent preparations of the calibration curves, each calibration curve consisted of five standard samples for a total of 25 independent standards. The %RSD ranged from 3.2 to 9.6% for PS80 and 1.1 to 2.4% for histidine. The calibration curves were constructed as shown in Figure 2C and D with *r* ^2^ = 0.9998 for PS80 and *r* ^2^ = 0.9999 for histidine standard curves and slope %RSD was 3.5% for PS80 and 1.3% for histidine (*n* = 5).

### 3.2 Analysis of HPV VLP samples

PS80 and histidine were measured in HPV VLP samples and the results are shown in Table 1 and compared with corresponding orthogonal methods: colorimetric for PS80 and RP HPLC for histidine. The data show that histidine concentrations agree between NMR and RP HPLC methods, however, the PS80 concentration measured in NMR trended slightly lower compared to the colorimetric assays. To investigate and further understand the apparent lower trending observed in the NMR method, the PS80 reference standard used in colorimetric assays was tested using NMR and it gave identical results as a reference standard used in NMR. Furthermore, a spike recovery experiment was performed in NMR. PS80 was spiked into an HPV buffer and HPV VLP sample that did not contain PS80. Both buffer and sample measured by NMR resulted in a 100% recovery. The data suggest that there is no loss of PS80 due to either surface adsorption or binding to VLP, which agrees with what was previously observed by Shi et al., demonstrating that PS80 does not bind to HPV VLP [24]. The cause of this bias was not determined, however, the PS80 assay variability (<20% for colorimetric and <10% for NMR) indicates the results are comparable and not statistically different between methods.

**TABLE 1:** PS80 and histidine

Lot #	% PS80 Colorimetric ^a^	% PS80 NMR^a^	mM Histidine RP HPLC^a^	mM Histidine NMR^a^
1	0.017	0.013	20.4	20.0
2	0.017	0.013	20.1	20.5
3	0.019	0.014	20.4	20.1
4	0.017	0.014	20.1	19.8
5	0.018	0.014	20.4	19.8
6	0.017	0.014	20.5	20.1
7	0.016	0.013	20.0	19.5
8	0.016	0.013	20.1	19.2
9	0.017	0.013	20.3	19.5
10	0.016	0.013	20.0	19.7

^a^ The %RSD of PS80 colorimetric is <20%, PS80 NMR is <10%, Histidine RP HPLC is <5%, and Histidine NMR is <5%.

%RSD is defined as (standard deviation/average) × 100%.

PS80 and histidine content in HPV VLP‐containing aluminum adjuvant sample was also measured using the NMR technique. It was prepared by mixing the HPV VLP aqueous product with the alum adjuvant in a 50:50 ratio and stored in a glass container at 2–8°C. The samples were centrifuged for 15 min at 15 K RPM and the supernatant was collected for NMR analysis. The results shown in Table 2 indicate that PS80 and histidine concentrations in the HPV VLP alum‐containing adjuvant are about twofold less than HPV VLP in aqueous buffer, which is expected result with the 50:50 dilution with the adjuvant. This data demonstrate that PS80 does not bind to the alum‐containing adjuvant. The new NMR method works in the final HPV formulation with and without aluminum adjuvant.

**TABLE 2:** PS80 NMR in HPV samples containing‐alum adjuvant

Lot #	% PS80 in aqueous	% PS80 in alum adjuvant	mM Histidine in aqueous	mM Histidine in alum adjuvant
7	0.013	0.0064	19.5	9.84
9	0.013	0.0064	19.5	9.84

### 3.3 Stability of PS80

**FIGURE 3:**
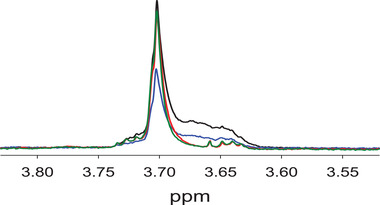
Comparison of NMR peak at 3.70 ppm of PS80 (0.015%) in histidine buffer stored in glass (blue trace) and stainless steel (red trace) containers. The PS80 containing HPV VLP sample stored in stainless steel (green trace) and PS80 reference standard control at 0.02% (black trace)

A buffer containing only PS80 and histidine was stored in glass and stainless steel containers for 2 months at 2–8°C. The NMR spectra are shown in Figure 3, illustrating the difference in PS80 NMR lineshape at 3.70 ppm between glass (blue trace) and stainless steel (red trace) containers. Similar results were observed for PS80 in the presence of HPV VLP stored in stainless steel (green trace). The PS80 results from colorimetric and NMR assays are shown in Table 3, illustrating that there is about a 70% decrease in PS80 concentration in stainless steel containers measured by colorimetric method. However, no difference was observed for the PS80 concentration in either the stainless steel or glass container measured by NMR. The NMR measures total protons in polyoxyethylene group, and NMR method was unable to determine quantitative concentration differences in stability stressed samples (Table 3). These data indicate the number of protons are not different in stressed samples, but qualitatively a line shape profile difference is evident (Figure 3). These data suggest that the NMR method cannot quantitatively be used for this stability studies, but can still show for qualitative line shape differences. Similar observation and conclusion were reported for PS20 quantitation using NMR by Khossravi et al. [25].

**TABLE 3:** PS80 in HPV buffer stored in glass versus stainless steel containers

Container	Lot #	% PS80 NMR	% PS80 Colorimetric
Glass	A	0.010	0.010
Stainless steel	A	0.012	0.0032
Glass	B	0.016	0.016

**FIGURE 4:**
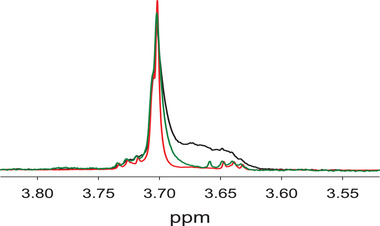
NMR peak at 3.70 ppm of PS80‐treated with NaOD (red trace) and compared with PS80‐containing HPV VLP stored in stainless steel (green trace) and PS80 reference standard as control (black trace)

The PS80 NMR line shape in stainless steel is much sharper than in glass containers by an apparent increase in resolution and losing the broad shoulder peak. This suggests that the hydrophilicity of polyoxyethylene is removed from the hydrophobic tail of the oleic acid and there is also a loss of heterogeneity, due to the fact that various types of fatty acid tails are being cleaved, hence, it is capable of freely rotating in solution. When it is attached to the hydrophobic tail, it tumbles slowly in NMR time scale due to the micelle form of PS80 and, hence, its line shape is broad due to slight anisotropy. To investigate this hypothesis, base‐catalyzed hydrolysis in PS80 ester linkage was performed by adding NaOD. Figure 4 shows that the change of PS80 line shape at 3.70 ppm can be closely reproduced after adding NaOD into PS80 (red trace) as compared without NaOD but stored in stainless steel (green trace). Although it is not exactly the same profile, the overall loss of the broad shoulder peak is apparent (see Supporting Information for time‐dependent PS80 hydrolysis by NaOD). The results suggest that PS80 stored in a stainless steel container causes its degradation in ester‐linkage likely through metal‐catalyzed oxidation, among other potential degradation mechanisms as previously observed by Gopalrathnam et al. [3].

## 4 CONCLUDING REMARKS

A relatively fast and simple NMR method to determine histidine and PS80 concentration in the presence of an HPV VLP sample was developed. This one method can potentially be used to replace two independent methods of measuring histidine by RP HPLC and PS80 by iodine‐starch colorimetry, hence, increasing efficiency during product development. Furthermore, the NMR method can detect the PS80 stability by observing its polyoxyethylene line shape region. Although this is a qualitative analysis, it will be a much faster evaluation of PS80 stability as compared to any other methods. We have developed an NMR method using a high‐field 600 MHz NMR spectrometer, next we plan to conduct additional experiments to determine if this method can be transferred to a simpler benchtop low‐field 60 MHz NMR for routine use in quality control laboratories. Additional challenges for 60 MHz benchtop NMR include lower sensitivity and a broader line width that can cause overlap between the polyoxyethylene and α− or β‐histidine proton peaks. These challenges will be evaluated.

## CONFLICT OF INTEREST

The author has declared no conflict of interest.

## Supporting information

Supporting information

## Data Availability

The data that support the findings of this study are available from the corresponding author upon reasonable request.
