# Supplementary material for: Polysorbate 80 and histidine quantitative analysis by NMR in the presence of virus‐like particles
Source: Electrophoresis. 2022 Apr 26;43(13-14):1408–14. doi: 10.1002/elps.202100189 (PMC9544792; doi:10.1002/elps.202100189)
Supplement: Supplementary file 1 — Supporting information [file ELPS-43-1408-s001.pptx]

## Slide 1
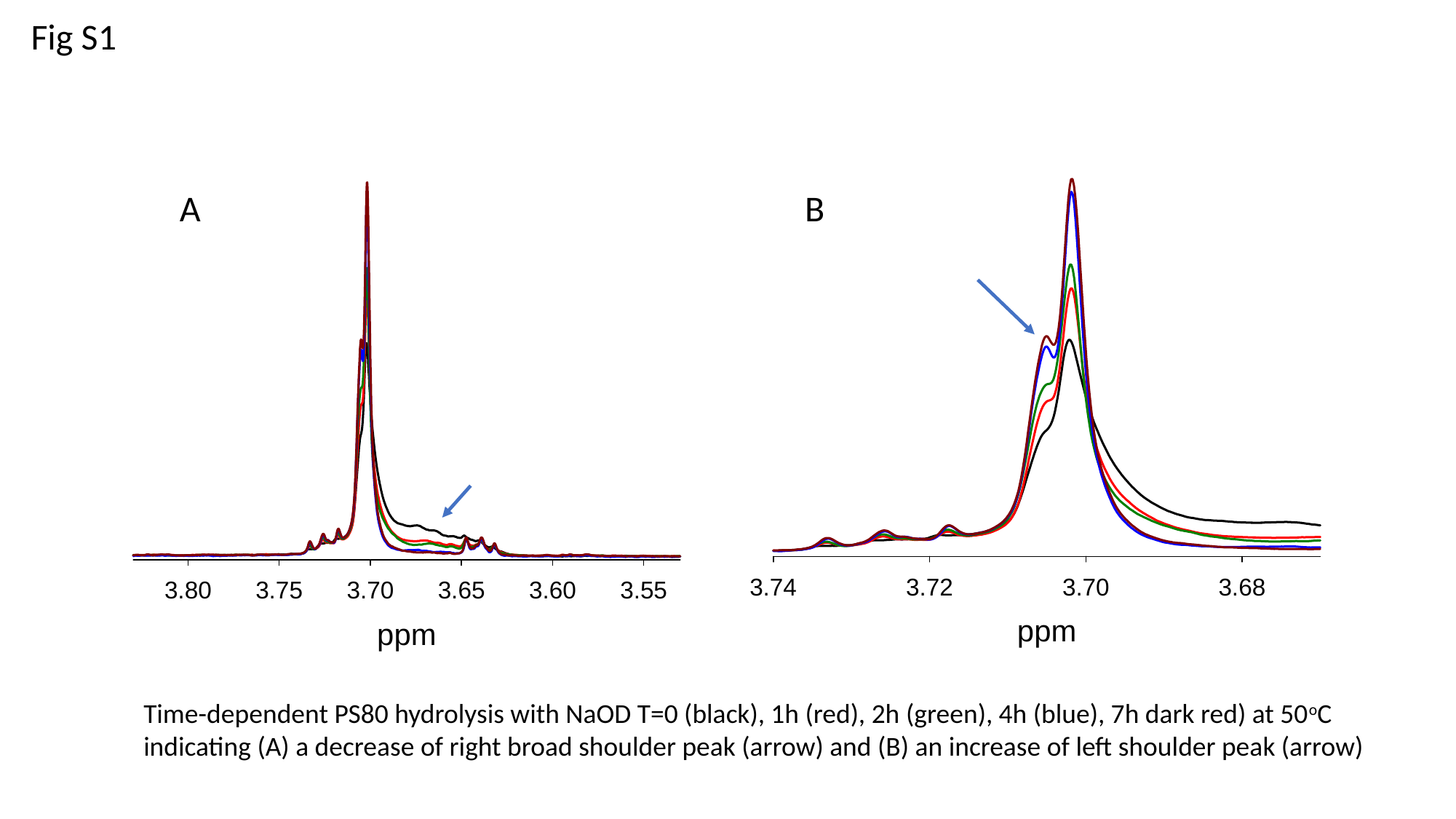

Fig S1
A
B
Time-dependent PS80 hydrolysis with NaOD T=0 (black), 1h (red), 2h (green), 4h (blue), 7h dark red) at 50oC
indicating (A) a decrease of right broad shoulder peak (arrow) and (B) an increase of left shoulder peak (arrow)

## Slide 2
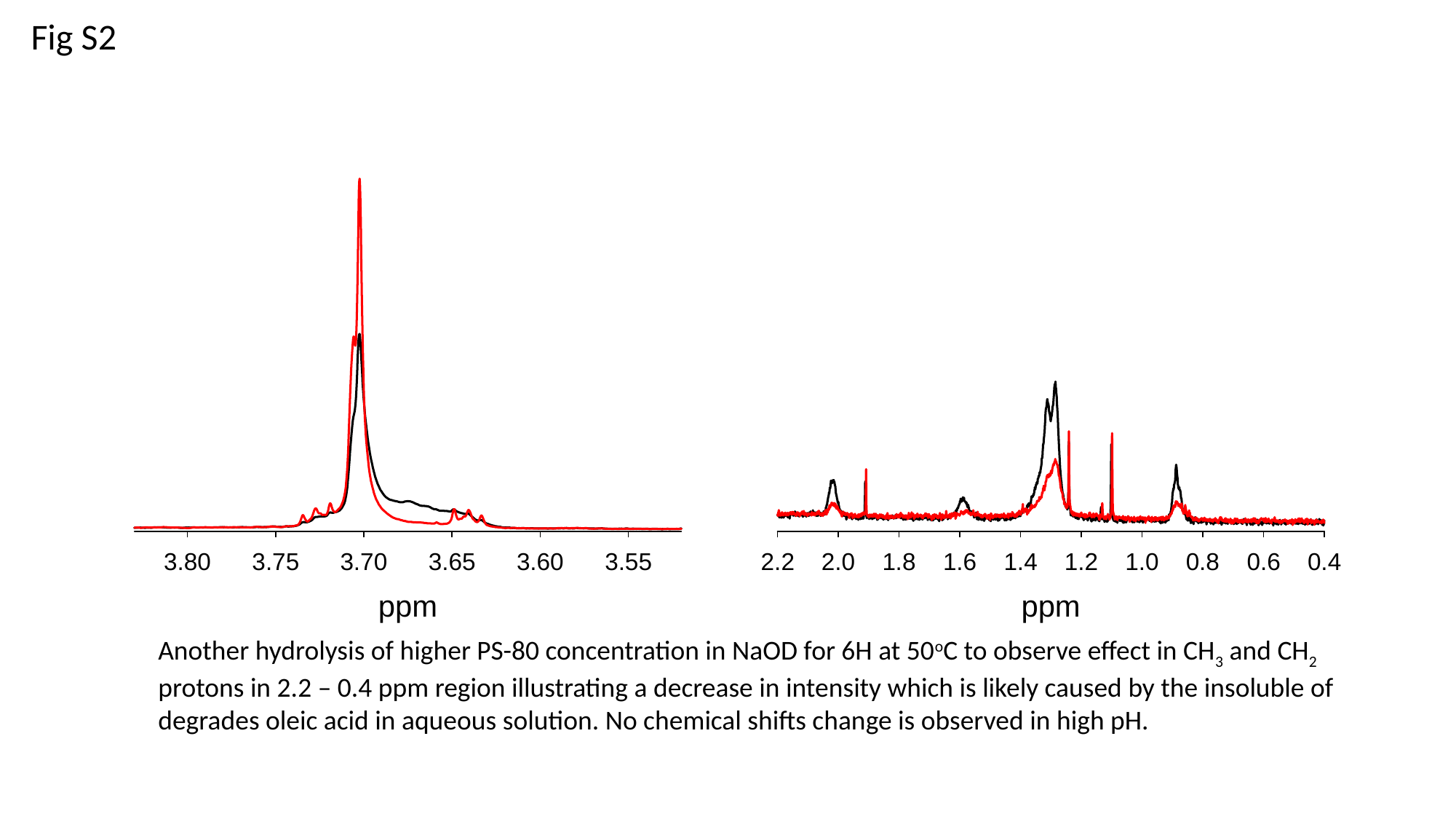

Fig S2
Another hydrolysis of higher PS-80 concentration in NaOD for 6H at 50oC to observe effect in CH3 and CH2
protons in 2.2 – 0.4 ppm region illustrating a decrease in intensity which is likely caused by the insoluble of
degrades oleic acid in aqueous solution. No chemical shifts change is observed in high pH.

## Slide 3
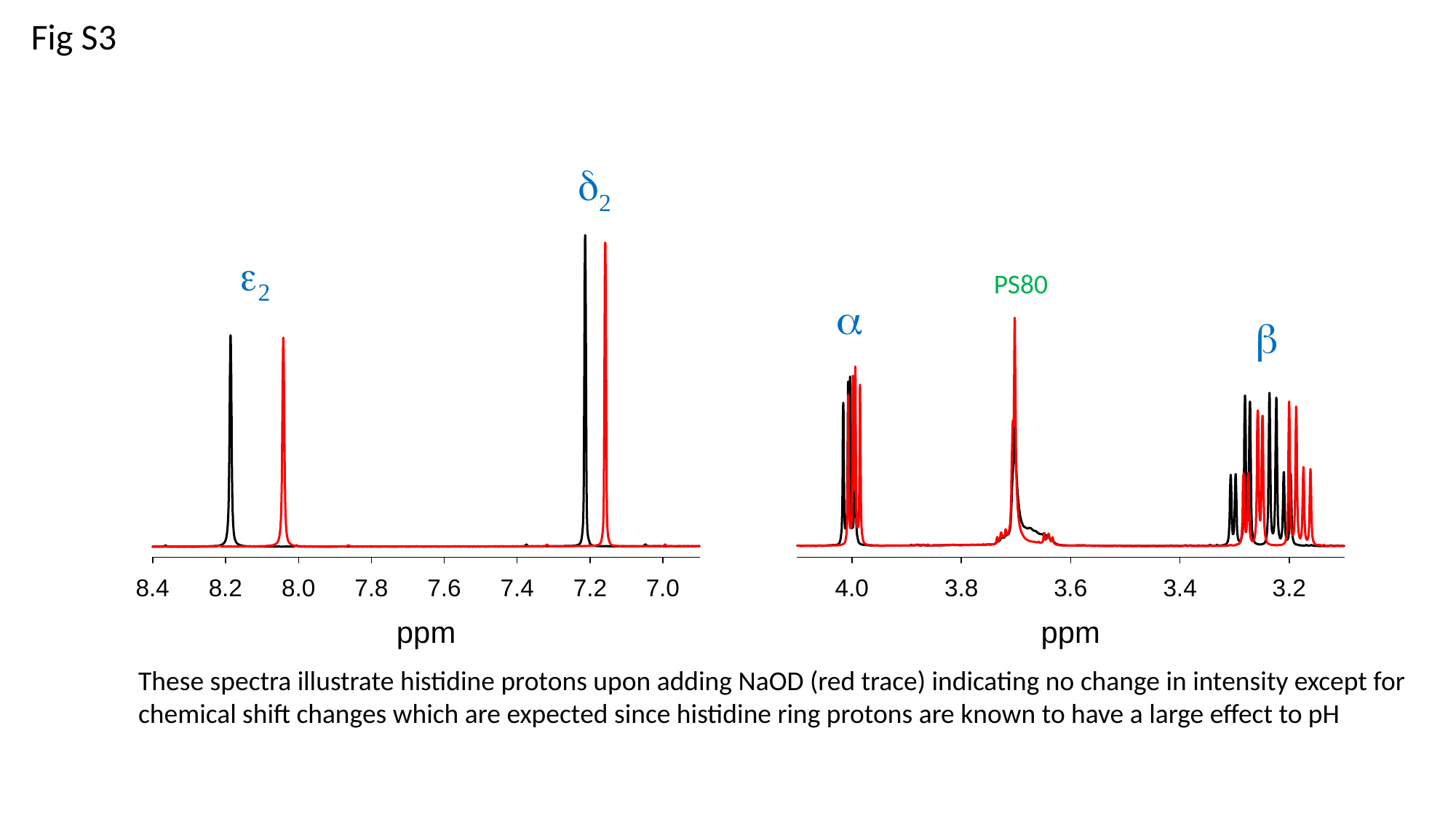

Fig S3
d2
e2
PS80
a
b
These spectra illustrate histidine protons upon adding NaOD (red trace) indicating no change in intensity except for
chemical shift changes which are expected since histidine ring protons are known to have a large effect to pH
